# Introduction of gasless laparoscopic surgery as a minimally invasive procedure for endometrial cancer and its usefulness from the viewpoint of the learning curve

**DOI:** 10.1186/s12957-021-02453-6

**Published:** 2021-12-18

**Authors:** Hiroe Ito, Tetsuya Moritake, Fumitoshi Terauchi, Keiichi Isaka

**Affiliations:** 1 Department of Obstetrics and Gynecology, Tokyo Medical University Hospital, 6-7-1, Nishishinjuku, Shinjuku-ku, Tokyo, 160-0023 Japan; 2 Department of Obstetrics and Gynecology, Sugawara Hospital, Koshigaya, Japan; 3 Robotic Surgery Center, Tokyo International Ohori Hospital, Tokyo, Japan

**Keywords:** Endometrial cancer, Gasless laparoscopic surgery, Learning curve, Uterine manipulator, Survival rate

## Abstract

**Background:**

We investigated the usefulness of gasless laparoscopic surgery (GLS) using a subcutaneous abdominal wall lifting method for endometrial cancer.

**Methods:**

We studied 105 patients with early endometrial cancer who underwent GLS (55) or open surgery (50). A uterine manipulator was used in all GLS cases. We compared operative time, blood loss, number of lymph nodes removed, hospital stay, perioperative complications, cases converted to laparotomy, and recurrence and survival rates. We also studied the learning curve and proficiency of GLS.

**Results:**

The GLS group had significantly longer operative time (265 vs. 191 min), reduced blood loss (184 vs. 425 mL), shorter hospital stay (9.9 vs. 17.6 days), and fewer postoperative complications (1.8 vs. 12.0%) than the open group. No case was converted to laparotomy. Disease-free and overall survival rates at 4 years postoperatively (GLS vs. open groups) were 98.0 versus 97.8 and 100 versus 95.7%, respectively, and there was no significant difference between the groups. Regarding the learning curve for GLS, two different phases were observed in approximately 10 cases. Operator 2, who was not accustomed to laparoscopic surgery, showed a significant reduction in operative time in the later phase 2.

**Conclusions:**

GLS for endometrial cancer results in less bleeding, shorter hospital stay, and fewer complications than open surgery. Recurrence and survival rates were not significantly different from those of open surgery. This technique may be introduced in a short time for operators who are skilled at open surgery but not used to laparoscopic surgery.

## Introduction

Endometrial cancer is the second most frequent gynecologic malignancy after cervical cancer and has been on the rise recently. In Japan, endometrial cancer accounts for more than 30% of all uterine cancers, and its incidence has more than doubled in the past 10 years [1].

The treatment methods include surgery, chemotherapy, radiation therapy, and hormone therapy, but surgery is the most common treatment for early stage endometrial cancer. In terms of surgery, total abdominal hysterectomy, bilateral adnexectomy, and lymph node dissection have been performed for a long time, but in recent years, laparoscopic surgery has become popular due to advances in endoscopic equipment

Laparoscopic surgery for endometrial cancer is less painful than open surgery and patients can be discharged earlier with less pain and fewer postoperative complications [2, 3]. In addition, the oncologic outcome reportedly is no different from that of open surgery [4]. On the other hand, laparoscopic surgery requires advanced technology to perform using forceps with limited movement in the limited space of the abdominal cavity. Furthermore, problems exist, such as complications and port site metastasis, caused by insufflation [5, 6].

The subcutaneous abdominal wall lifting (SAWL) method has been used to avoid complications caused by the pneumoperitoneum [7, 8]. Unlike the pneumoperitoneum method, the operative field with SAWL is secured by elevating the abdominal wall. Since no gas is used, it also is called gasless laparoscopic surgery (GLS). The advantages of GLS include excellent safety because there are no complications caused by the pneumoperitoneum, such as carbon dioxide absorption and nerve reflex. Also, the operative field is not obstructed by suction and smoke emission, and suturing and ligating are very easy. The use of conventional surgical instruments minimizes the use of disposable products, which is very economical [9]. Finally, because there is no need to use pistol-type forceps during laparoscopic surgery, which are difficult to operate, the proficiency of the procedure is excellent. The disadvantage of GLS is that the operative field is narrower than that achieved with a pneumoperitoneum because there is no exclusion of the intestinal tract due to insufflation pressure. Since we introduced SAWL to laparoscopic surgery in 1994 [10], we have consistently improved this method, aiming for a laparoscopic surgical procedure that many surgeons can master easily [11].

We introduced GLS using SAWL with improved disadvantages while maintaining the advantages of GLS during early endometrial cancer surgery. We examined its usefulness compared to open surgery.

## Materials and methods

### Study cases

Of 105 patients diagnosed with International Federation of Gynecology and Obstetrics (FIGO) stage IA endometrial cancer by histopathologic and diagnostic imaging examination at Tokyo Medical University Hospital, 55 (GLS group) underwent GLS between July 2014 and May 2017, while 50 (control group) underwent laparotomy between March 2009 and December 2014. Sufficient informed consent about the operation was obtained. This study was approved by the Institutional Review Board of the University (T-2020-0077).

### Procedure to secure the operative field:

**Fig. 1:**
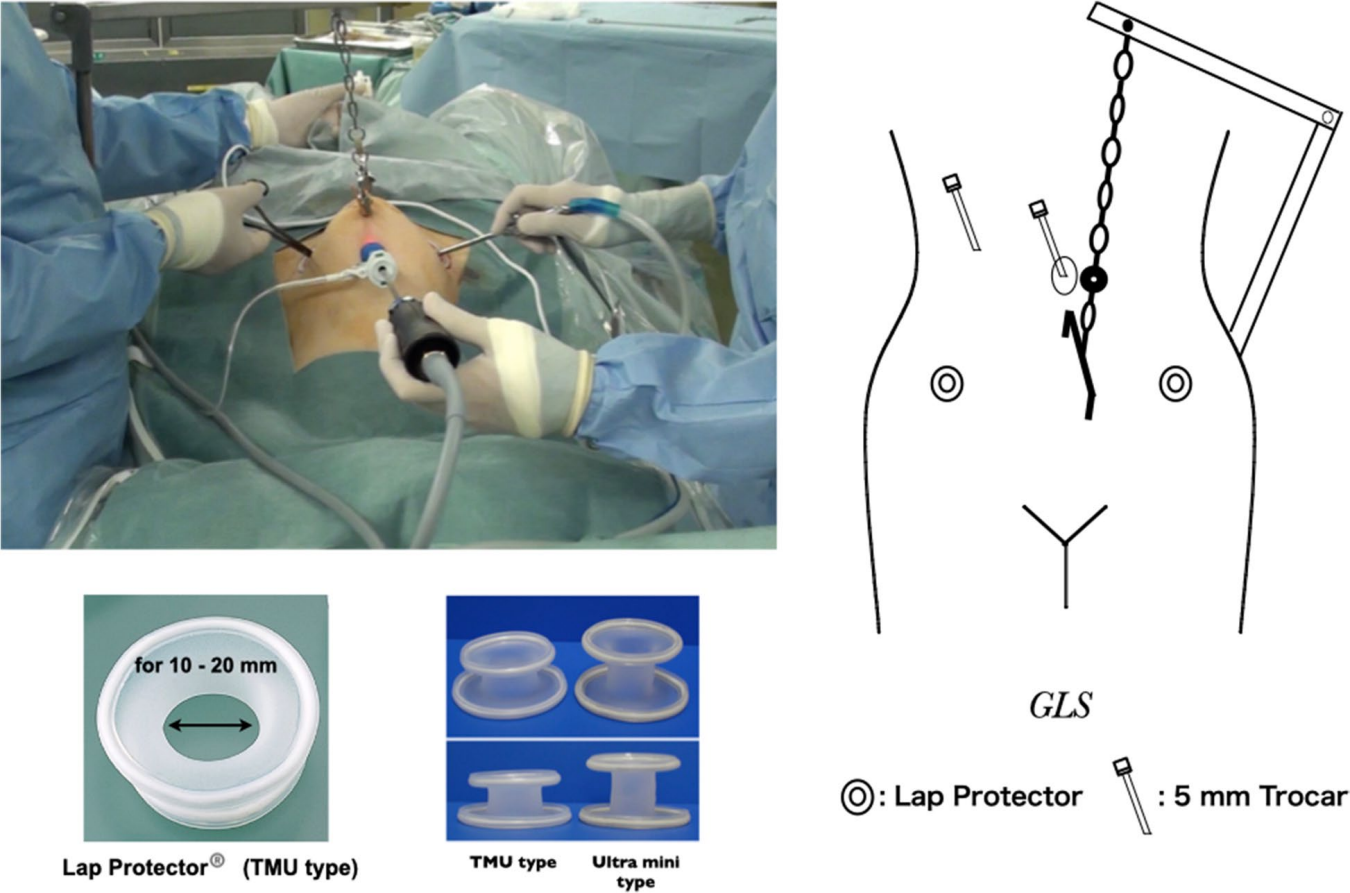
Photograph and schematic diagram of the gasless laparoscopic surgery (GLS) using the subcutaneous abdominal wall lifting (SAWL) procedure. The photograph shows the surgeon inserting two forceps from the lap protector to perform surgery. The assistant controls the camera and grasps the tissue with the forceps. The schematic shows the location of lifting equipment, Lap Protectors, and ports. The Lap Protector is usually the smallest Tokyo Medical University (TMU) type. This corresponds to a port size of 10–20 mm. In addition, for obese cases with a thick abdominal wall, a tall ultra mini type is used

The operative field was secured via SAWL (Fig. 1). SAWL was performed using the simple lifting instruments from Mizuho Medical Co., Ltd. (Tokyo, Japan). Briefly, a 1.2-mm Kirschner steel wire was inserted subcutaneously into the mid-abdominal wall of the lower abdomen and fixed to the lifting handle. The abdominal wall then was raised by hooking the chain of the lifting handle to the lifting bar. A small 1.5-cm incision was made in the right abdominal wall of the patient with a scalpel to create a port, and the Lap Protector® (Hakko, Chikuma, Nagano, Japan) was inserted and installed. A 5-mm trocar was punctured into the umbilical fossa under endoscopic monitoring to form an endoscope port. After creating a port on the left abdominal wall in the same way as on the right side, the Lap Protector was inserted and installed. If necessary, a 5-mm trocar was punctured on the right abdomen at the level of the navel for use with a grasping forceps. The patient was in the 30° Trendelenburg position, and a uterine manipulator was used in all cases. An ultrasonic surgical aspirator (SonoSurg; Olympus, Center Valley, PA, USA) was used as an auxiliary for lymph node dissection.

### The surgeons

The 55 GLS procedures were performed by 10 surgeons (including five gynecologic oncologists) with sufficient experience in open surgery for endometrial cancer. No surgeon had previous experience with laparoscopic surgery plus pelvic lymphadenectomy for endometrial cancer.

Of these cases, 24 were operated by a surgeon proficient in laparoscopic surgery as well as open surgery (Operator 1) and 17 by a surgeon proficient in open surgery but were novice to laparoscopic surgery (Operator 2). Operator 1 has performed more than 500 cases of laparoscopic surgery, while Operator 2 performed only eight. On the other hand, open surgeries were performed by 15 surgeons (including six gynecologic oncologists) experienced with uterine cancer surgery, and the gynecologic oncologist always participated in the operation as the operator or first assistant.

### Operation method

**Fig. 2:**
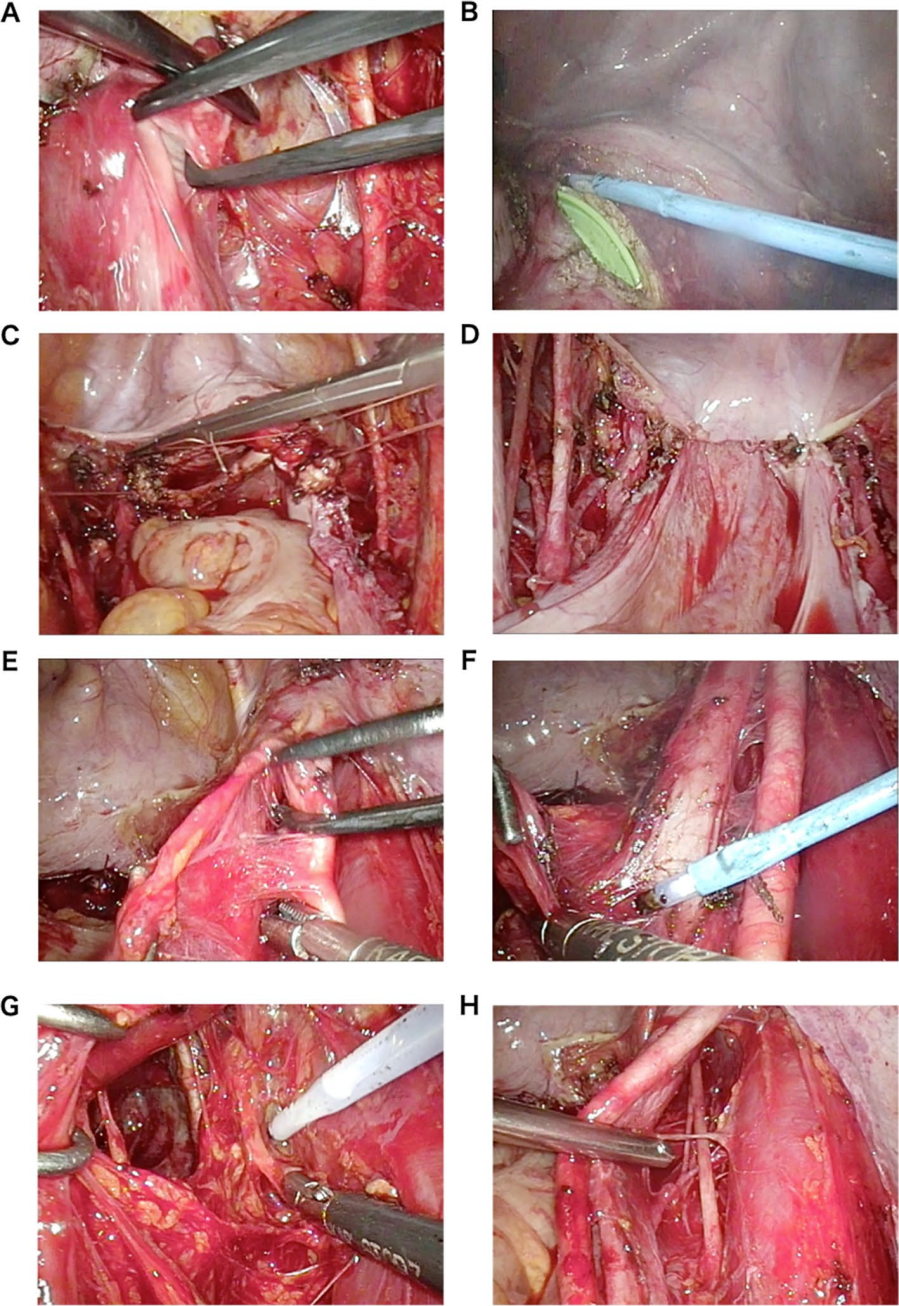
Hysterectomy (**A** to **D**) and right pelvic lymph node dissection (**E** to **H**) by this procedure. **A** Development of ureteral tunnel. **B** Vaginal incision using an electrocautery. **C** Suture of vaginal stump using needle holder (single nodule suture). **D** Completion of peritoneal suture at the vaginal stump. **E** Exfoliation of the external iliac artery. **F** Exfoliation of lymphatic fat mass from external iliac vein. **G** Dissection using SonoSurg. **H** Lymph node dissection around the obturator nerve

Basically, total hysterectomy, bilateral adnexectomy, and pelvic lymphadenectomy were performed (Fig. 2), but lymphadenectomy was omitted in some cases.

### Comparative study between two groups

Patient background, operative method, operative time, blood loss, hospital stay, perioperative complications, numbers of conversions to laparotomy, postoperative chemotherapy, and recurrence and survival (overall [OS] + disease-free [DFS]) rates, as well as postoperative pathologic diagnoses were compared in both groups.

### Comparative study on proficiency of GLS

To examine the proficiency of GLS for endometrial cancer, we compared the surgical results achieved between Operators 1 and 2, such as the operative time, blood loss, number of lymph nodes removed, hospital stay, and weight of the removed uterus. However, both operators had extensive experience with pelvic lymphadenectomy during open surgery, but no experience with laparoscopic pelvic lymphadenectomy.

### Statistical analysis

The comparison between the groups was evaluated using the Student *t* test, and the groups were correlated using the *χ*^2^ test. DFS and OS survival curves were prepared using the Kaplan–Meier method, and a statistical comparison between the groups was performed using a log-rank test. *P* < 0.05 was considered statistically significant. Statistical analysis was performed using the Statistical Package for the Social Sciences version 25 (SPSS 25; SPSS, Inc., Chicago, IL, USA).

The learning curve of GLS for endometrial cancer was evaluated using cumulative sum (CUSUM) analysis [12, 13]. The CUSUM value for the operative time of the first case was obtained by calculating the difference between the operative time and average operative time of all cases. As for the CUSUM value after the first case, the value of the previous case was added to the value obtained by the difference from the average operative time. For example, in the second case, the CUSUM value of the first case was added to the difference between the operative time of the second case and the average operative time of all cases. A continuous graph was obtained by calculating this to the last case.

## Results

### Patient background and surgical results

The mean age and mean body mass index in the GLS group were not significantly different from values in the open group (54.7 ± 10.8 vs. 58.0 ± 13.6 years [*P* = 0.170] and 23.1 ± 5.1 vs. 24.7 ± 5.8 [*P* = 0.133], respectively). The main operation method was total hysterectomy and pelvic lymph node dissection in 89.1% and 88.0%, respectively, cases overall, and 10.9% and 12.0%, respectively, among cases in which lymph node dissection was omitted. There was no bias in surgical procedure between the two groups (*P* = 0.861, Table 1).

**Table 1:**
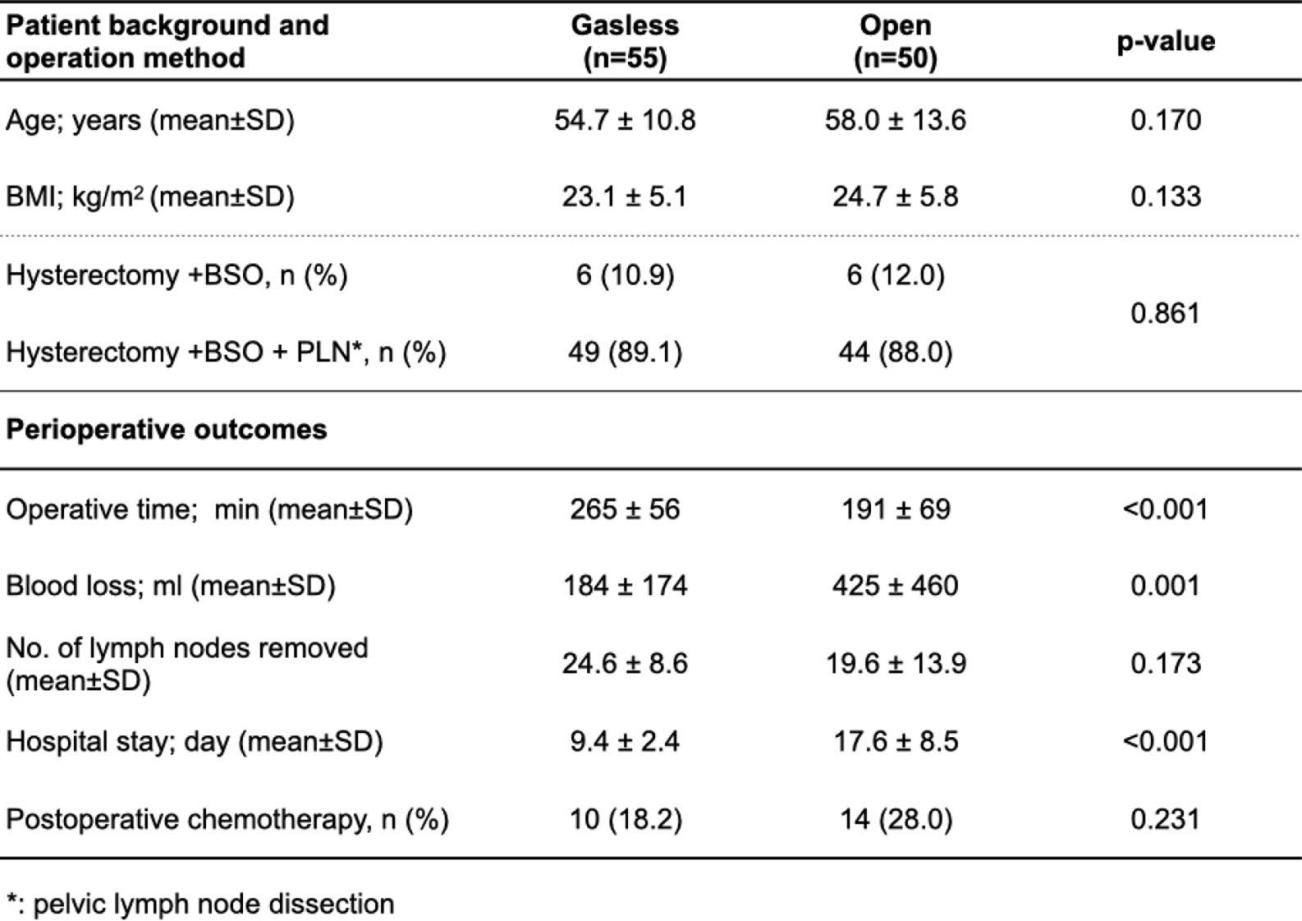
Patient background and operation methods, and perioperative outcomes in both groups

Regarding surgical results, the GLS group showed a significant increase in operative time (265 vs. 191 min) compared to the open group (*P* < 0.001), but blood loss was significantly reduced (184 vs. 425 mL; *P* = 0.001) and hospital stay was significantly shortened (9.4 vs. 17.6 days; *P* < 0.001). No significant difference was found in the number of lymph nodes removed (24.6 vs. 19.6; *P* = 0.173). Ten patients (18.2%) in the GLS group and 14 (28.0%) in the open group received chemotherapy postoperatively (no significant difference, *P* = 0.231, Table 1).

Perioperative complications occurred in three (5.5%) and eight (16.0%) cases, respectively (no significant difference, *P* = 0.078). Intraoperative complications were found in two GLS group patients, but none in the open group (no bias was observed, *P* = 0.173).

On the other hand, postoperative complications of Clavien-Dindo classification grade II or higher were observed in one case (1.8%) in the GLS group and six (12.0%) in the open group (significant difference in both groups, *P* = 0.032, Table 2). The main complications were wound dehiscence, ileus, and fever. In addition, none of the GLS procedures was converted to laparotomy.

**Table 2:**
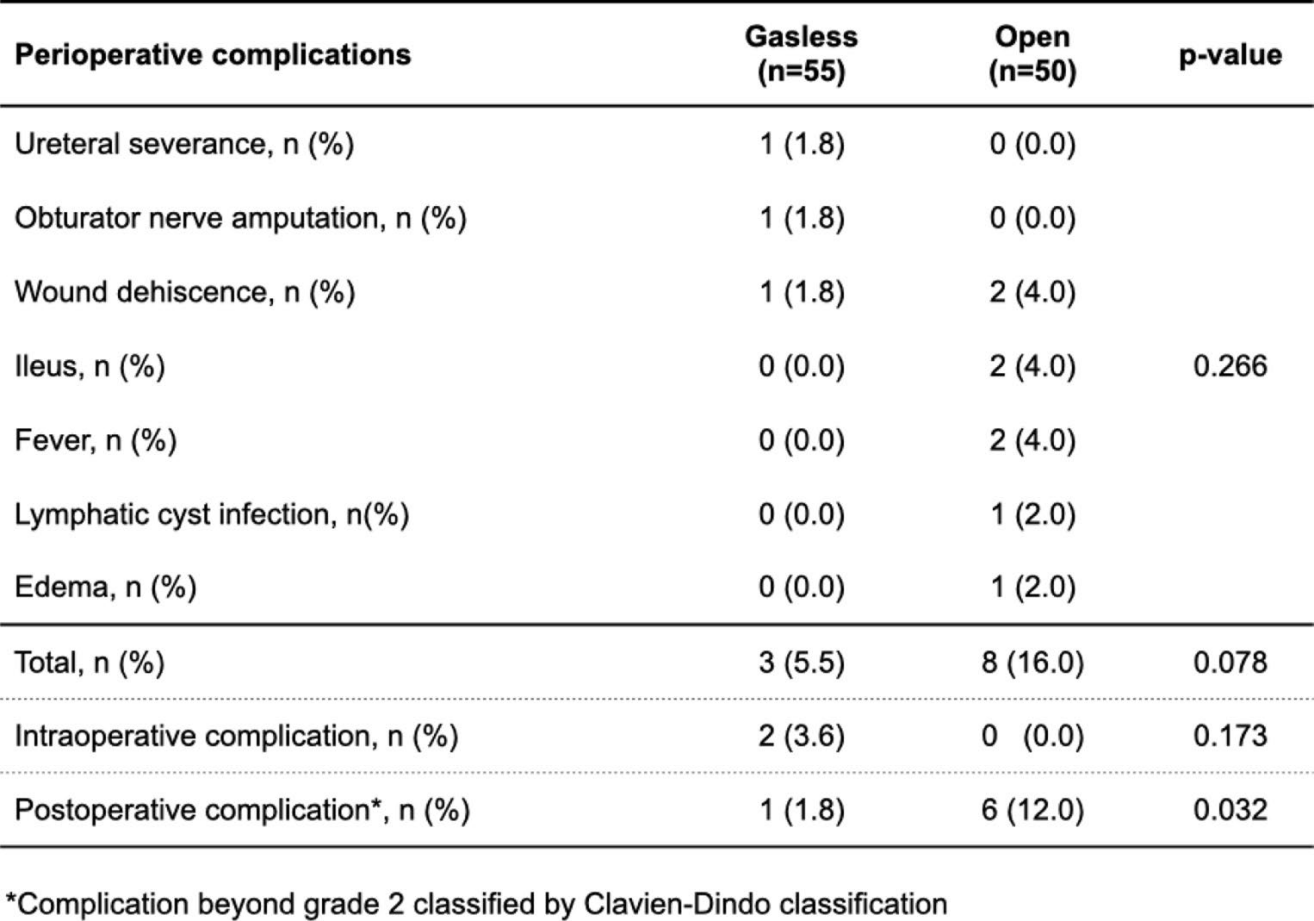
Comparison of perioperative complications between both groups

### Comparison of postoperative pathologic diagnosis

The postoperative pathologic diagnosis in the GLS group was FIGO stage lA in 50 patients (90.9%), stage IB in 3 (5.5%), and stage III in 2 (3.6%). On the other hand, in the open group, 43 patients had stage 1A (86.0%), 1 stage 1B (2.0%), 3 stage I (6.0%), 1 stage I (2.0%), and 2 stage II (4.0%) disease. No significant bias was observed in the groups (*P* = 0.156, Table 3).

**Table 3:**
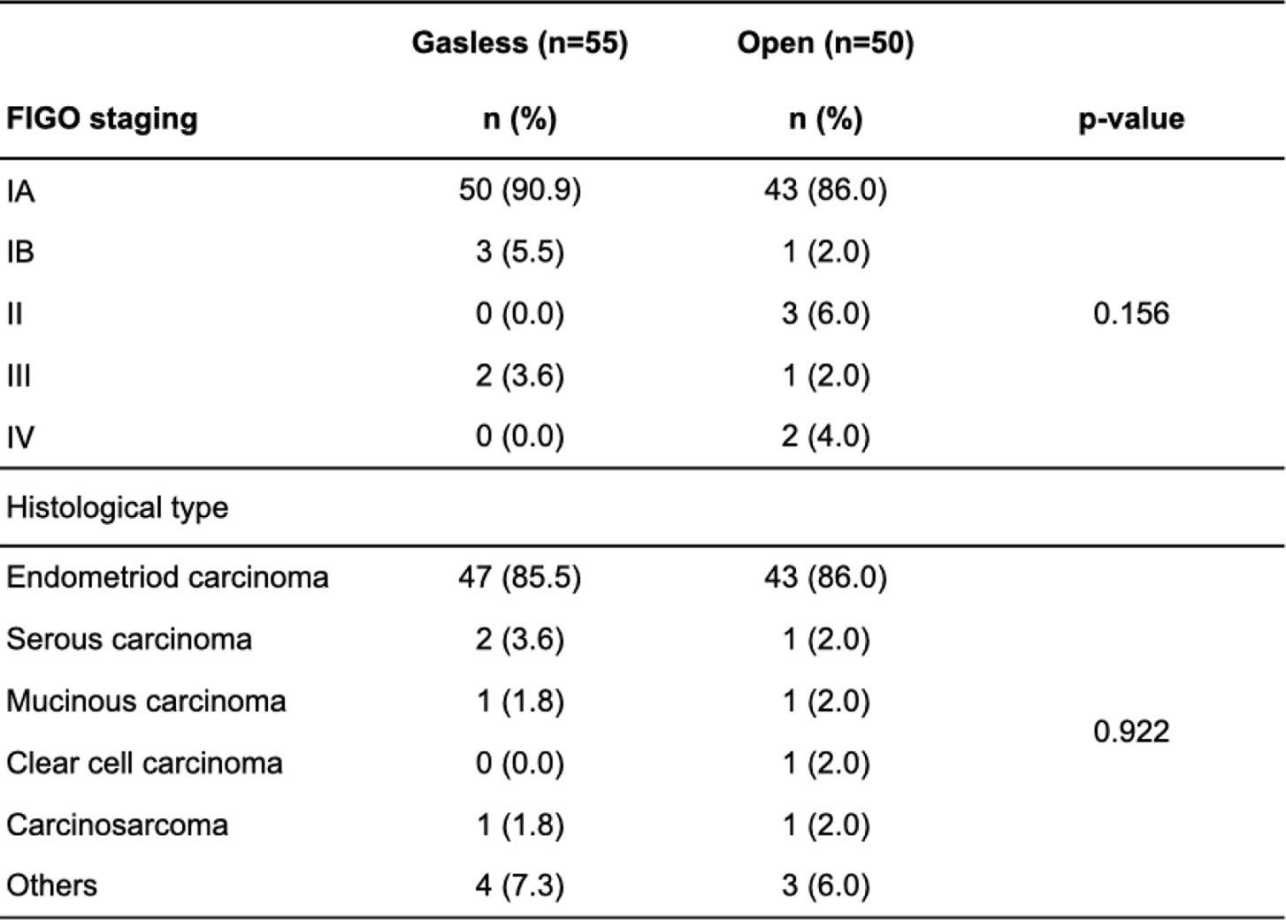
Comparison of FIGO staging and postoperative histologic type between both groups

In the GLS group, endometrioid carcinoma was the most common histologic type in 47 cases (85.5%), followed by serous carcinoma in 2 (3.6%), mucinous carcinoma and carcinosarcoma in 1 each (1.8%), and other types in 4 (7.3%). On the other hand, in the open group, endometrioid carcinoma was the most common in 43 cases (86.6%), followed by serous carcinoma, mucinous carcinoma, clear carcinoma, and carcinosarcoma in 1 each (1.3%), and other types in 3 (6.0%). There was no significant bias between the groups (*P* = 0.922, Table 3).

### Oncologic outcomes

**Fig. 3:**
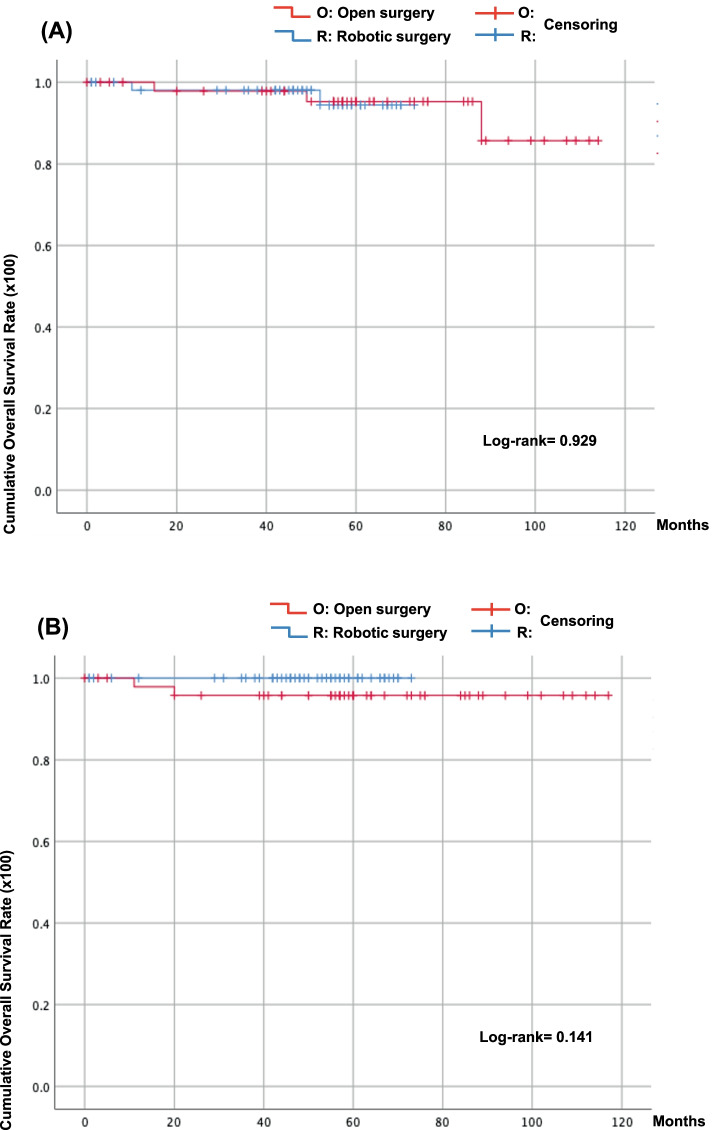
Kaplan–Meier curves of disease-free survival (DFS), log-rank test *P*= 0.929 (**A**) and overall survival (OS), log-rank test *P*= 0.141 (**B**). *Tick marks* indicate censored data. The differences in survival between the groups were not significant

Mean follow-up postoperatively was 48.2 ± 17.9 months in the GLS group and 62.8 ± 29.1 months in the open group. DFS at 4 years was 98.0% and 97.8%, respectively. On the other hand, OS at 4 years was 100% and 95.7%, respectively. There were no significant differences between the groups with respect to DFS and OS (Fig. 3).

All recurrences were in FIGO stage I cases (two [3.6%] in the GLS and three [6.0%] in the open groups; no significant difference, *P* = 0.570, Table 4).

**Table 4:**
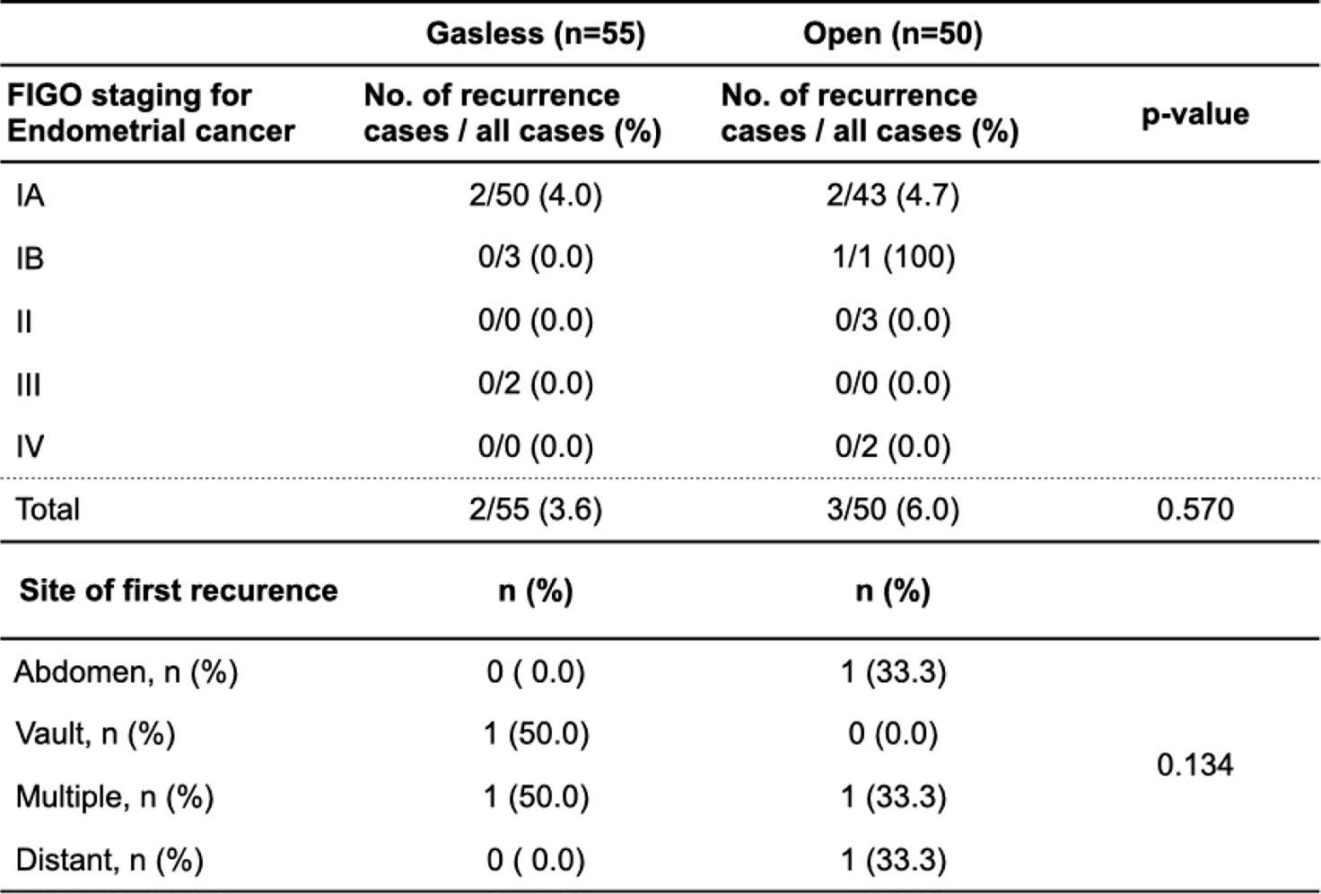
Operative outcomes. Rate of recurrence by FIGO staging and site of the first recurrence in both groups

Regarding the site of recurrence, one case each was in the vault and multiple sites in the GLS group, and one in the open group was in the abdomen, multiple sites, and distant. There was no significant bias in recurrence sites between the groups (*P* = 0.134, Table 4).

### A study on proficiency of GLS

**Fig. 4:**
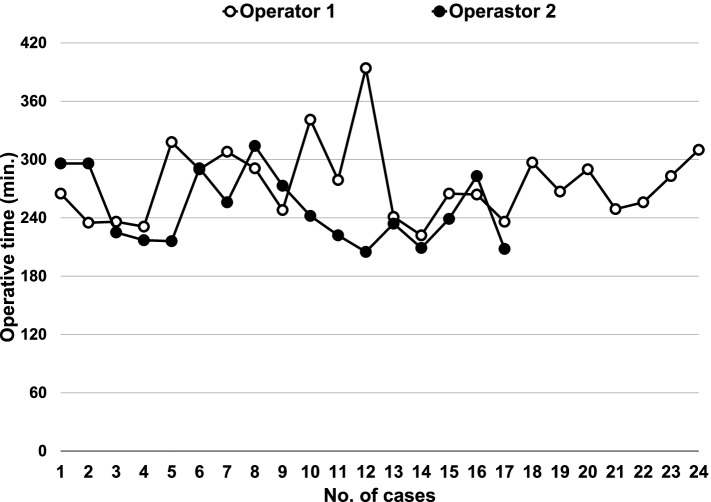
Comparison of learning curves for the raw operative time plotted against chronologic case number between both surgeons

**Fig. 5:**
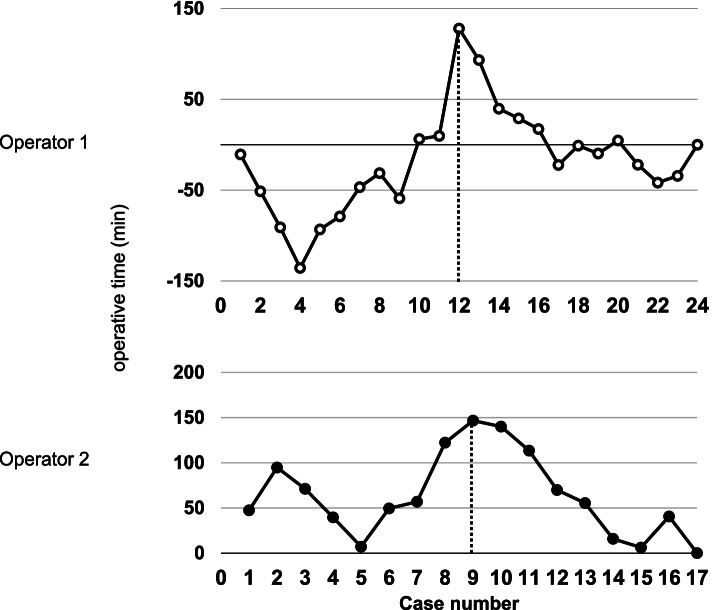
Cumulative sum (CUSUM) learning curves for operative time plotted against chronologic case number by both surgeons

Figure 4 shows the learning curves for the raw operative time plotted against chronologic case number by both surgeons. The learning curve for operative time of Operators 1 and 2 as an index showed two different phases (phase 1 showed a positive slope and phase 2 showed a negative slope). The turning point was the case 12 for Operator 1 and the case 9 for Operator 2. Operator 2 showed a significant reduction in operative time in the later phase 2 (Fig. 5, Table 5).

**Table 5:**
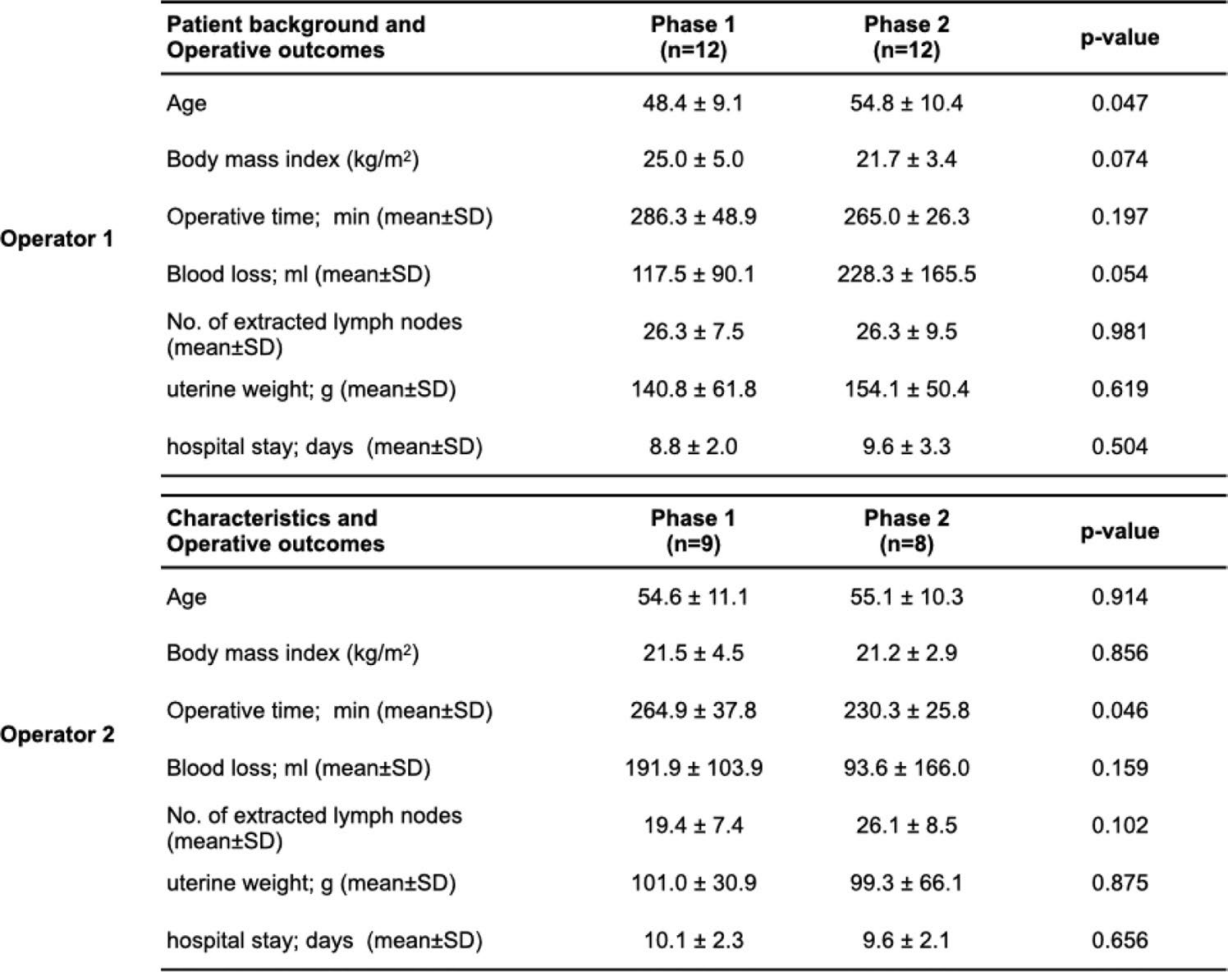
Comparison of patient background and operative outcomes between phases of both surgeons (Operators 1 vs 2)

Furthermore, when comparing both surgeons, no significant difference was observed between them regarding operative time (*P* = 0.059), blood loss (*P* = 0.548), number of lymph nodes removed (*P* = 0.172), and hospital stay (*P* = 0.394). However, the weight of the removed uterus was significantly higher for Operator 1 compared to Operator 2 (*P* = 0.019, Table 6).

**Table 6:**
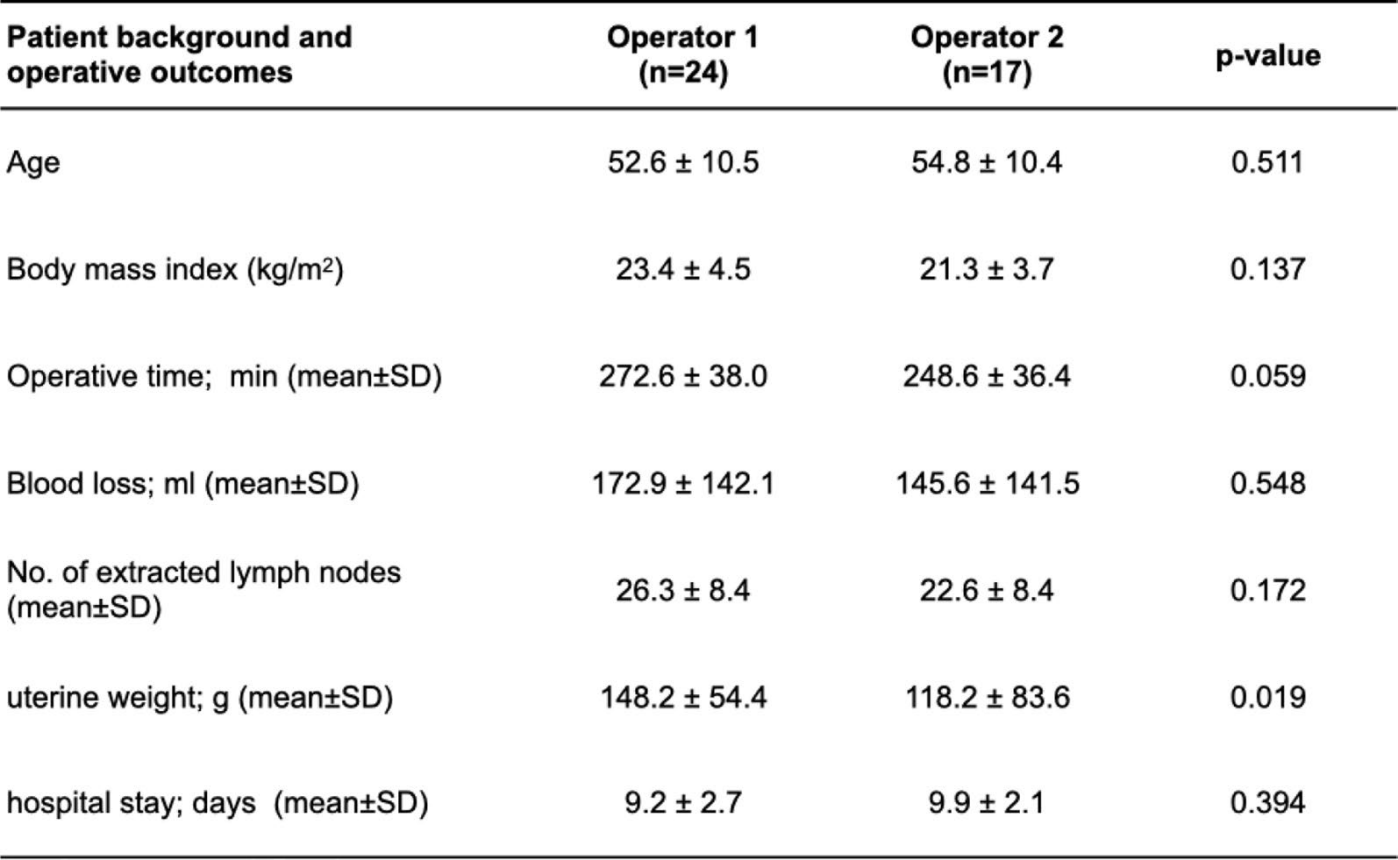
Comparison of patient background and operative outcomes between both surgeons (Operators 1 vs 2)

## Discussion

Laparoscopic surgery has been introduced in various departments for malignant tumor surgery, and the results are comparable to those of open surgery [14–16]. Laparoscopic surgery for endometrial cancer has many advantages because it is minimally invasive, and the oncologic outcome reportedly is not inferior to that of open surgery [4, 17]. Presently, laparoscopic surgery is becoming a gold standard procedure for endometrial cancer instead of open surgery.

On the other hand, pneumoperitoneum may have adverse effects on cardiac and respiratory diseases, and the effects of pneumoperitoneum on malignant tumors such as port-site metastasis are still unknown [6, 18, 19]. Therefore, in this study, we introduced GLS without insufflation into the surgery of endometrial cancer.

Endometrial cancer surgery using GLS reduced blood loss, shortened hospital stay, and reduced complications compared to open surgery. Complications in the GLS group were significantly less than those in the open group, but this probably was due to less postoperative ileus and wound dehiscence as reported in laparoscopic surgery using pneumoperitoneum [20]. On the other hand, two cases of intraoperative complications were observed in the GLS group, but these were believed due to inexperienced forceps operation in the initial introduction stage.

Since these results were the same result as that achieved by conventional laparoscopic surgery with pneumoperitoneum [21, 22], GLS may be useful as a minimally-invasive procedure for endometrial cancer.

In GLS, the surgeon may be forced to operate in a narrower operative field than with conventional insufflation. Therefore, we attempted to compensate for this deficiency by using a steep Trendelenburg position (30°) and a uterine manipulator. With this approach, we were able to perform surgery without much concern about intestinal tract interference with the operative field. In the future, we would like to further improve the surgery by using instruments such as the 3-dimentional endoscope [23].

Regarding the use of the uterine manipulator, there are two contradictory reports: in one report, intraperitoneal tumor cells increased due to contact between the manipulator and tumor [24, 25] and in one the tumor cells did not increase [26, 27]. However, the use of uterine manipulators reportedly had no effect on oncologic outcomes [28, 29]. In our study, we showed statistically that use of a uterine manipulator did not affect recurrence or survival rates. However, since the average observation period was as short as 41 months, a longer observation period is necessary in the future.

On the other hand, since GLS results in no operative field disorder due to abdominal collapse caused by gas leakage, surgical procedures, such as lymph node dissection using an ultrasonic surgical aspirator, vaginal wall incision for hysterectomy, removal of the uterus from the vagina, and vaginal stump suture, were performed easily in a stable operative field. These procedures are difficult to perform when the pneumoperitoneum method is used, and this is considered an advantage of GLS.

Another advantage of GLS has been reported to be more proficient for beginners than the conventional laparoscopic surgery using pneumoperitoneum [30]. In the present study, the proficiency of GLS by two surgeons using CUCUM analysis was recognized as a biphasic learning curve with two phases: a positive slope (phase 1, representing the initial learning curve) and a negative slope (phase 2, representing the mastery phase that shortens operative time) [31, 32]. The turning points changing from phases 1 to 2 were found in the early cases before and after the tenth case in both operators. In particular, Operator 2, who was not accustomed to laparoscopic surgery, showed a significant reduction in operative time in phase 2, which indicated that GLS is easy to learn even for beginners in laparoscopic surgery. On the other hand, the fact that Operator 1 showed an operative time shorter than the average operative time from the beginning of phase 1 is probably because Operator 1 was sufficiently familiar with laparoscopic surgery. The positive slope after that time is presumed to be the result of trial and error in pursuit of a more reliable procedure as he became used to the technique.

In the comparative study between two surgeons, one a laparoscopic surgery expert and one a beginner, there was no significant difference in operative time, blood loss, and number of lymph nodes removed. We speculated that the proficiency level of laparoscopic surgery does not affect the learning of GLS. The significant difference in weight of the removed uterus between the two surgeons may be attributed to the fact that Operator 2 was a novice to laparoscopic surgery and, thus, technically avoided a large uterus.

In GLS, the surgeon can perform operations similar to those during open surgery using forceps. Thus, GLS has the advantage of easily performing procedures, such as lymphadenectomy and suturing of the vaginal stump. We believe that this factor makes it easy for beginners to learn laparoscopic surgery. Our study showed that GLS has a high proficiency even with highly difficult laparoscopic methods if the surgeon is sufficiently proficient at open surgery.

However, this study has some limitations. First, we relied on the experience of two surgeons rather than the results from evaluations of many surgeons. Next, when comparing the results of the two surgeons, no consideration was given to the port size. Unlike with a conventional pneumoperitoneum, in GLS, multiple forceps can be inserted and operated from one port, so the difficulty of surgery increased if the port size was small. Since Operator 1 had abundant experience with laparoscopic surgery, the port size was set as small as possible in consideration of cosmesis. Therefore, it is undeniable that there is a bias in the difficulty of their surgeries. In this study, there was no difference in patient background between the two phases, but if there is a difference in surgical difficulty depending on the case, the operative time may be biased and the learning curves of both may be affected.

In conclusion, GLS for early endometrial cancer resulted in less bleeding, shorter hospital stay, and fewer complications than open surgery, with no significant difference in recurrence and survival rates compared to open surgery. GLS may be an option in diseases where pneumoperitoneum is thought to have adverse effects. The results also suggest that GLS is a technique that can be introduced in a short time to surgeons who are skilled at open surgery but are not accustomed to laparoscopic surgery.

## Data Availability

Not applicable

## Abbreviations

GLS: Gasless laparoscopic surgery
SAWL: The subcutaneous abdominal wall lifting
FIGO: International Federation of Gynecology and Obstetrics
OS: Overall
DFS: Disease-free
SPSS: Statistical Package for the Social Science
