# Correction to: Introduction of gasless laparoscopic surgery as a minimally invasive procedure for endometrial cancer and its usefulness from the viewpoint of the learning curve

**DOI:** 10.1186/s12957-022-02491-8

**Published:** 2022-02-24

**Authors:** Hiroe Ito, Tetsuya Moritake, Fumitoshi Terauchi, Keiichi Isaka

**Affiliations:** 1 Department of Obstetrics and Gynecology, Tokyo Medical University Hospital, 6-7-1, Nishishinjuku, Shinjuku-ku, Tokyo, 160-0023 Japan; 2 Department of Obstetrics and Gynecology, Sugawara Hospital, Koshigaya, Japan; 3 Robotic Surgery Center, Tokyo International Ohori Hospital, Tokyo, Japan

**Correction to: World J Surg Onc 19, 347 (2021)**

**https://doi.org/10.1186/s12957-021-02453-6**

In this article [1] the wrong figure appeared as Fig. 5; the figure should have appeared as shown below.

**Fig. 5:**
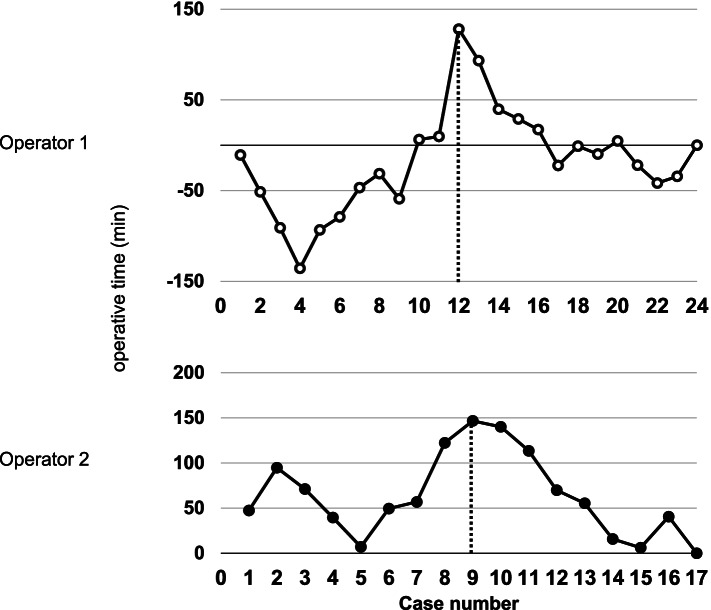
Cumulative sum (CUSUM) learning curves for operative time plotted against chronologic case number by both surgeons

The original article has been updated.

## References

[1] Ito H, Moritake T, Terauchi F (2021). Introduction of gasless laparoscopic surgery as a minimally invasive procedure for endometrial cancer and its usefulness from the viewpoint of the learning curve. World J Surg Onc.

